# Supplementary material for: Fabrication of PCDTBT Conductive Network via Phase Separation
Source: Materials (Basel). 2021 Sep 4;14(17):5071. doi: 10.3390/ma14175071 (PMC8433801; doi:10.3390/ma14175071)
Supplement: Supplementary file 1 [file materials-14-05071-s001.zip › materials-1349030-supplementary.pdf]

Supporting Information for

Fabrication of PCDTBT Conductive Network via Phase Separation

Jianwei Xu[*1], Zhiming Liu[1], Lei Jing[1], Jingbo Chen[1**]

[1]School of Materials Science & Engineering, Zhengzhou University, Zhengzhou 450002, People's Republic of China

Correspondng Author E-mail：

[*] *xujianwei@zzu.edu.cn (J. Xu),*

[**] *chenjb@zzu.edu.cn (J. Chen)*

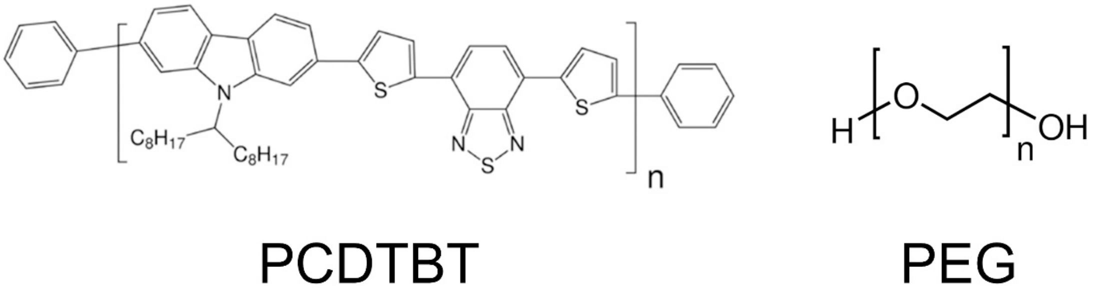

Figure S1. Molecular chemical structures of PCDTBT and PEG.

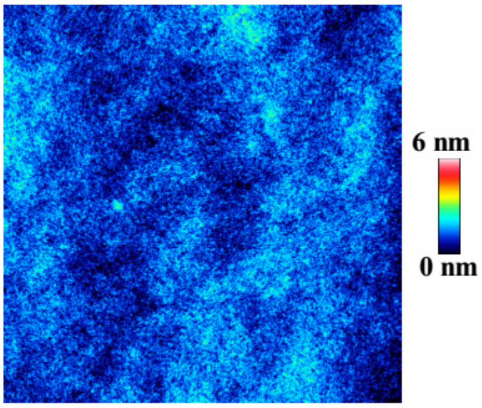

Figure S2. AFM height image of pure PCDTBT thin film without post treatment. The size of AFM image is 3 × 3 μm^2.

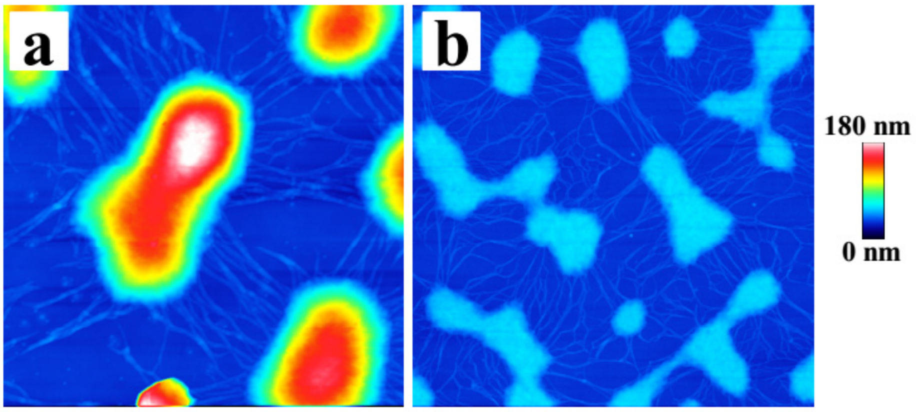

Figure S3. AFM height images of PCDTBT/PEG (weight ratio 1:1) the washed films correspond to blend films produced with solution concentration of (a) 3.0 wt-%, (b) and (c) 0.5 wt-%. The size of each AFM image is 3 × 3 μm^2.

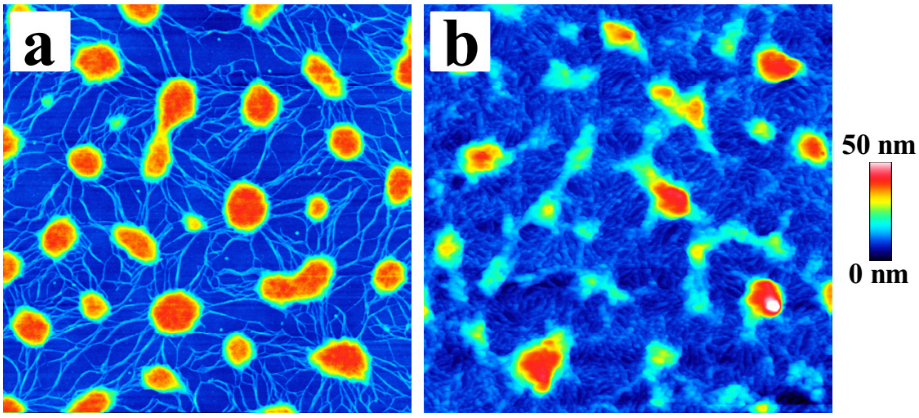

Figure S4. AFM height images of PCDTBT/PEG (weight ratio 4:6) the washed films correspond to blend films produced with PCDTBT molecular weight of (a) 16.2 kg/mol and (b) 30.0 kg/mol. The size of each AFM image is 3 × 3 μm^2.
